# NUPR1: A Critical Regulator of the Antioxidant System

**DOI:** 10.3390/cancers13153670

**Published:** 2021-07-22

**Authors:** Can Huang, Patricia Santofimia-Castaño, Juan Iovanna

**Affiliations:** Centre de Recherche en Cancérologie de Marseille (CRCM), INSERM U1068, CNRS UMR 7258, Aix-Marseille Université and Institut Paoli-Calmettes, Parc Scientifique et Technologique de Luminy, 163 Avenue de Luminy, 13288 Marseille, France; can.huang@inserm.fr (C.H.); patricia.santofimia@inserm.fr (P.S.-C.)

**Keywords:** NUPR1, cell death, ferroptosis, ROS, cell stress

## Abstract

**Simple Summary:**

Nuclear protein 1 (NUPR1) is activated in cellular stress and is expressed at high levels in cancer cells. Much evidence has been gathered supporting its critical role in regulating the antioxidant system. Our review aims to summarize the literature data on the impact of NUPR1 on the oxidative stress response via such a regulatory role and how its inhibition induces reactive oxygen species-mediated cell death, such as ferroptosis.

**Abstract:**

Nuclear protein 1 (NUPR1) is a small intrinsically disordered protein (IDP) activated in response to various types of cellular stress, including endoplasmic reticulum (ER) stress and oxidative stress. Reactive oxygen species (ROS) are mainly produced during mitochondrial oxidative metabolism, and directly impact redox homeostasis and oxidative stress. Ferroptosis is a ROS-dependent programmed cell death driven by an iron-mediated redox reaction. Substantial evidence supports a maintenance role of the stress-inducible protein NUPR1 on cancer cell metabolism that confers chemotherapeutic resistance by upregulating mitochondrial function-associated genes and various antioxidant genes in cancer cells. NUPR1, identified as an antagonist of ferroptosis, plays an important role in redox reactions. This review summarizes the current knowledge on the mechanism behind the observed impact of NUPR1 on mitochondrial function, energy metabolism, iron metabolism, and the antioxidant system. The therapeutic potential of genetic or pharmacological inhibition of NUPR1 in cancer is also discussed. Understanding the role of NUPR1 in the antioxidant system and the mechanisms behind its regulation of ferroptosis may promote the development of more efficacious strategies for cancer therapy.

## 1. Introduction

The human NUPR1 gene, located on chromosome 16, codes for a low molecular weight protein (8 kDa), NUPR1, which lacks a stable secondary and tertiary structure, and is expressed at low levels in healthy cells under normal physiological conditions [1]. However, NUPR1 can be transcriptionally activated under stress conditions such as oxidative stress, ER stress, and metabolic stress, to protect the cells from these stresses [2]. Compared with normal cells, cancer cells, particularly those in the tumor microenvironment, show higher basal levels of cellular stress such as oxidative stress and ER stress [3,4]. Thus, NUPR1 is present at correspondingly higher levels in cancer cells [5]. Oxidative stress refers to elevated levels of ROS that damage biomacromolecules such as proteins, DNA, and lipids in cells [6]. In order to protect themselves from such ROS-mediated tumor cell damage or cell death, cancer cells up-regulate the antioxidant system [7]. In cancer cells, ER stress may activate the ubiquitin–proteasome system (UPS), the unfolded protein response (UPR), or other cytoprotective mechanisms to restore homeostasis and increase the adaptability to the adjacent environment [8,9,10]. Methamphetamine (METH)-induced ER stress has been shown in non-cancer cells to initiate apoptosis and autophagy via the NUPR1/CHOP/TRIB3 pathway [11,12,13]. Interestingly, ER stress can be triggered by high levels of ROS, which emphasizes the role of NUPR1 in the crosstalk between different stress responses [14]. Moreover, increased aerobic glycolysis and oxidative stress are important features of metabolism in cancer cells [15]. NUPR1 is a highly expressed protein that confers drug resistance to cancer cells through the maintenance of redox and the antioxidant system in various pathological conditions [16,17]. Increasing evidence shows that NUPR1 is activated under high ROS levels to protect cells against oxidative damage. Recently, ferroptosis, an oxidative cell death mechanism, was discovered in V-Ki-ras2 Kirsten rat sarcoma viral oncogene homolog (KRAS) mutant tumor cells, and is characterized by antioxidant defense system failure and the induction of lipid peroxidation [18]. Ferroptosis is the result of a redox imbalance caused by the cancer cells’ incapacity to maintain cell metabolism and cellular functions though antioxidant defense [19]. It does not occur frequently due to the strong antioxidant defense mechanism present in cancer cells [20]. Previously, the GSH/GPX4 system was thought to be the only regulator of ferroptosis [21,22]. Later, more signals were found to suppress ferroptosis, such as COQ10/FSP1 [23,24] and BH4/DHFR [25]. The mounting evidence supporting ferroptosis as a therapeutic target has led to the development or identification of more ferroptosis inducers for cancer treatment [26]. They have proven useful in overcoming drug resistance and preventing tumor metastasis formation [27], and may represent a promising strategy in combination with other anti-cancer therapies. One important process in cancer cells allowing the formation of drug resistance and heterogeneity is the upregulation of NUPR1 expression [28]. The NUPR1 was recently shown to participate towards iron and energy metabolism and protect cells or tissues against ferroptosis [29,30]. Nevertheless, how NUPR1 is transcriptionally activated in the oxidative stress response and its exact role in ferroptosis remain unclear. In this review, we aimed to clarify the role of NUPR1 in the antioxidant system and redox balance. We provide arguments favoring the targeting of NUPR1 to induce ferroptosis as a therapeutic strategy for treating cancer.

## 2. Oxidative Stress Activates NUPR1

NUPR1 expression is associated with the deregulation of ROS levels in cells, and has been shown to be promoted with elevated ROS levels. For example, by increasing ROS levels, quercetin can induce the expression of NUPR1 and activates autophagy in osteosarcoma cells [31]. Either supplementation of the antioxidant N-acetyl cysteine (NAC) or knockout of NUPR1 can inhibit the autophagy and lipid peroxidation induced by quercetin, thus implicating the ROS/NUPR1 pathway in the mechanism used by quercetin [31]. Similarly, the cytotoxic heavy metal cadmium (Cd) can decrease glutathione (GSH) levels, causing oxidative stress and lipid peroxidation in cancer cells [32,33]. In an oral squamous carcinoma xenograft model, the Cd led to lipid peroxidation and induced NUPR1-dependent autophagy, which was again inhibited by NAC [34]. However, how ROS regulates the expression of NUPR1 in this context remains an open question.

### 2.1. Oxidative Stress Regulates NUPR1 Expression via a ER Stress-Mediated Pathway

**Figure 1:**
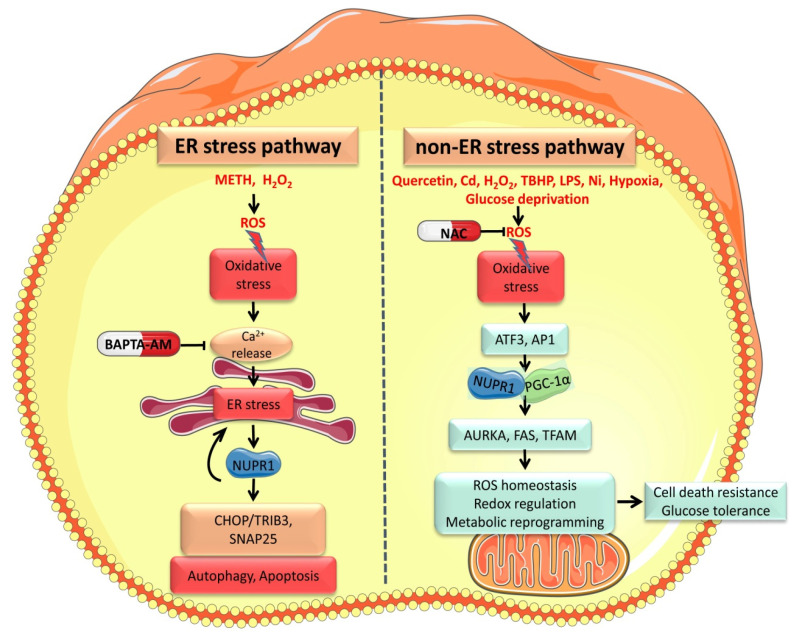
Oxidative stress activates NUPR1 by both ER stress (METH [11] and H_2_O_2_ [40]) and non-ER stress pathways (Quercetin [31], Cd [34], H_2_O_2_ [40,41], TBHP [42,43], LPS [44], Ni [45], hypoxia [46,47] and glucose deprivation [46,47,48].

NUPR1 can be transcriptionally activated under oxidative stress or ER stress conditions. ROS as well as both oxidants and reducing agents have been shown to disrupt protein folding and induce calcium release from the ER, leading to ER stress [35,36]. For example, intracellular ROS is sufficient to trigger the calcium ions (Ca^2+^) release from the ER, inactivate Ca^2+^-dependent ER partners (calnexin and calreticulin), and induce the ER stress response [14,37,38]. Concordantly, NUPR1 is activated in the ER stress response caused by intracellular high ROS levels. For example, the neurotoxic drug methamphetamine (METH) causes mitochondrial dysfunction, increases oxidative stress, changes intracellular Ca^2+^ dynamics, and thereby activates the ER stress response [39]. Xu and colleagues showed that apoptosis and autophagy induced by METH in pheochromocytoma are the consequence of the upregulation of NUPR1 during ER stress [11]. METH also activates the downstream pathways (CHOP/Trib3) of NUPR1 in ER stress, inhibits the mechanistic target of rapamycin (mTOR) phosphorylation, and thereby promotes neuronal autophagy [12]. Thus, Nupr1/CHOP/Trib3 signaling appears as a potential therapeutic target for drug-induced neurotoxicity. The involvement of Ca^2+^ is supported by evidence of the calcium chelator BAPTA-AM significantly inhibiting elevated NUPR1 mRNA levels in liver cancer cells treated with hydrogen peroxide (H_2_O_2_) [40]. Altogether, these results imply that the transcriptional activation of NUPR1 regulated by ROS is related to the ER stress triggered by Ca^2+^ (Figure 1).

### 2.2. Oxidative Stress Mediates NUPR1 Expression through Non-ER Pathways

Several studies have shown that the elevated NUPR1 level in oxidative stress is likely to be directly regulated by some factors in the non-ER stress pathway. For example, a ferroptosis inducer tert-butyl hydroperoxide (TBHP) can activate NUPR1 through the oxidative stress pathway, but not the ER stress pathway [42,43]. High intracellular ROS levels caused by H_2_O_2_ activate the expression of activating transcription factor 3 (ATF3), which then binds to the NUPR1 promoter to activate transcription [41]. Another study showed that NUPR1 was not activated in liver cancer cells exposed to H_2_O_2_ for a short time, but that NUPR1 protein levels increased over time accompanied by mitochondrial defects [40]. NUPR1 levels seem therefore to positively correlate with the ROS levels. In support of this notion, ROS and the NUPR1 were decreased in HERV-K env knockout colorectal cancer, compared with the wild-type cells; however, HERV-K re-expression restored the elevated levels of both NUPR1 and ROS [49]. Although ER stress can be initiated by ROS accumulation, ROS or Ca^2+^ release is insufficient to induce a severe ER stress response [50]. We recently found that the eukaryotic initiation factor 2α (eIF2α) phosphorylation and its downstream signaling pathways in ER stress require the participation of NUPR1 [51]. Indeed, our previous studies have shown that many proteins that participate in ER stress show minimal activation in the absence of NUPR1 [52]. While inhibition of NUPR1 significantly increases the ROS production, it dramatically inhibits the ER stress response, indicating that NUPR1 is a key mediator of the crosstalk between oxidative stress and ER stress (Figure 1).

## 3. NUPR1 Controls Redox Homeostasis and Protects Mitochondria

NUPR1 is a functional cellular stress protein which plays an important role in controlling its downstream signaling pathways and redox reactions. For example, oxidative damage to mitochondrial DNA and mitochondrial dysfunction caused by exposure of cells to the ferromagnetic metal nickel (Ni) [53], is associated with activated NUPR1 transcription through activator protein 1 (AP-1) binding to the NUPR1 promoter, in human bronchial epithelial cells. The NUPR1 thereby promotes the transformation of healthy cells into cancer cells as a protective mechanism against oxidative stress [45]. Knockdown of NUPR1 significantly reduced the cell viability and clonogenic ability of human bronchial epithelial cells exposed to Ni [45]. Therefore, high NUPR1 levels protect against oxidative damage, especially in those cells such as cancer cells with high ROS levels.

### 3.1. NUPR1 Regulates the Antioxidant System

The nuclear factor erythroid 2-related factor 2 (Nrf2) is an important transcription factor that regulates cellular oxidative stress response, and is also a central regulator that maintains intracellular redox homeostasis [54]. Nrf2 can reduce oxidative damage by regulating the constitutive expression of several antioxidant genes, and maintaining cellular redox homeostasis [55]. The NUPR1-mediated antioxidant pathway is, however, independent of NRF2 in the antioxidant system. Indeed, knockdown of NUPR1 triggers the expression of lipid detoxification genes such as aldo-keto reductase family 1 member C1 (AKR1C1) in keratinocytes and pancreatic cancer cells but does not affect NRF2 expression or nuclear translocation [56]. Inversely, knockdown of NRF2 showed no effect on the expression of either NUPR1 or AKR1C [56]. In another study, NUPR1 transcriptionally activated the presynaptic ROS sensor synaptosome associated protein 25 (SNAP25) and maintained the autolysosomal efflux in breast cancer cells [57,58]. In short, these studies emphasize the unique role of NUPR1 in the antioxidant system.

### 3.2. NUPR1 Maintains Mitochondrial Function

As the center of energy and the source of most ROS, mitochondria play an indispensable role in redox reactions [59]. The latest research shows that NUPR1 is involved in the regulation of mitochondrial function [40]. NUPR1 expression is usually induced in cells suffering persistent oxidative damage and mitochondrial dysfunction. For example, fascaplysin, a selective cyclin-dependent kinase 4 inhibitor with antitumor activity, can directly trigger mitochondrial depolarization and ATP consumption [60]. Studies showed that cell death induced by fascaplysin is accompanied by mitochondrial dysfunction, which causes the decreased mitochondrial membrane potential (MMP) and high ROS levels, and promotes NUPR1 expression as a protective defense [61]. However, knockdown of NUPR1 reduced the mitophagy induced by fascaplysin, and promoted greater ROS production [61]. These results indicate that the overexpression of NUPR1 is a protective process when mitophagy occurs in cells. In fact, NUPR1 inactivation causes mitochondrial dysfunction, antioxidant system failure, and accelerates ROS accumulation. Our previous study also showed that the inactivation of NUPR1 induced mitochondrial dysfunction including Ca^2+^ outflow, decreased ATP levels, and ROS increase in pancreatic cancer cells [52,62]. At the same time, the inactivation of NUPR1 reduced the expression of the tricarboxylic acid cycle (TCA) genes and activated glycolysis genes, thereby promoting a strong metabolic reprogramming in cancer cells [52].

### 3.3. NUPR1 Regulates Energy Metabolism

Mitochondria are the hub of energy metabolism, mainly using the energy from glucose, glutamine and fatty acid [63]. NUPR1 regulates cell energy metabolism by maintaining or enhancing the mitochondria function through different signal pathways. For instance, γ-H2AX, a histone that regulates ROS-mediated DNA damage through the Nox1/Rac1 pathway, was highly increased in NUPR1 knockdown cells subjected to hypoxia or glucose starvation [46,47,64]. The re-expression of NUPR1 or supplementation with NAC prevented oxidative DNA damage and reduced the γ-H2AX levels. Inhibition of aurora kinase A (AURKA), a gene associated with DNA damage in autophagy and that regulates mitochondrial dynamics and energy production, causes oxidative stress and subsequent ferroptosis in gastrointestinal cancer cells [65,66]. Intriguingly, hypoxia or glucose starvation can cause a decrease in AURKA levels with an increase in NUPR1, while knockdown of NUPR1 further significantly reduces the transcription and expression levels of AURKA, indicating that the overexpression of NUPR1 offsets the transcription changes during metabolic stress [46]. Glucose starvation also activates the molecular chaperones glucose-regulated protein 75 (GRP75) and glucose-regulated protein 94 (GRP94) because of a glycosylation defect-induced ER stress [67]. Elsewhere, NUPR1 has been shown to act as a co-activator of peroxisome proliferator-activated receptor-gamma coactivator 1-alpha (PGC-1α), a master regulator of ROS scavenging enzymes and an effective stimulator of mitochondrial biogenesis and respiration [68]. PGC-1α is responsible for the transcription of mitochondrial transcription factor A (TFAM), thereby maintaining the replication and transcription of mitochondrial genes [69]. The overexpression of PGC-1α leads to changes in the expression of the PGC-1α responsive gene fatty acid synthase (FAS) in prostate cancer [70]. Altogether, these studies demonstrate that NUPR1 regulates a series of signaling molecules to maintain mitochondrial function and energy metabolism.

In vivo studies have also underlined the important role of NUPR1 in energy metabolism [71,72]. For example, while wild-type mice showed impaired glucose tolerance under a short-term high-fat diet (HFD), NUPR1 knockout mice maintained normal glucose tolerance even after 16 weeks of HFD [48]. Elsewhere, compared with wild-type mice, NUPR1 knockout mice have increased β cell mass and higher insulin secretion by pancreatic islets [73]. Another study showed that following the HFD diet, NUPR1 knockout mice showed a significantly increased mass of islets, total number of islets, and average islet size, each indicative of protective measures against obesity, glucose intolerance, and insulin resistance [71]. These studies seem to indicate that NUPR1 knockout mice have stronger glucose tolerance and stronger glucose metabolism, suggesting that NUPR1 regulates energy metabolism in vivo.

Together, these data highlight the importance of high NUPR1 levels in maintaining energy metabolism and the antioxidant system. Compared with healthy cells, cancer cells have higher ROS levels due to their uncontrolled metabolic capacity during hyperproliferation [74,75]. The inactivation of NUPR1 in cancer cells can trigger ROS overproduction by inducing mitochondrial dysfunction, thereby leading to cell death [52]. One such potent inhibitor of NUPR1 is ZZW-115, which causes mitochondrial damage, energy metabolism deregulation, and ROS overproduction in a variety of cancer cells [62,76]. Altogether, NUPR1 exerts a protective role against oxidative damage by maintaining mitochondrial function and redox reactions in cells with high ROS levels.

## 4. NUPR1 Is a Key Factor of Ferroptosis

Increasing evidence suggests that the inactivation of NUPR1 impairs mitochondrial function and energy metabolism in cancer cells, increases ROS levels, and triggers a variety of cell death pathways, including apoptosis, autophagy, and necroptosis [77]. An increase in intracellular ROS acts as a primary signal that can react with intracellular iron to generate a more active ROS type-hydroxyl radical (HO•), thereby triggering ferroptosis [78,79]. Elsewhere, various ferroptosis inducers, such as erastin, RSL3 and sorafenib, were found to strongly activate NUPR1, supporting its protective role against ferroptosis [17,27].

### 4.1. NUPR1 Regulates Multiple Transcription Factors Involved in Ferroptosis

Evidence suggests the involvement of NUPR1 in ferroptosis through a variety of regulatory pathways. For example, RNF113A knockout dramatically reduced the NUPR1 overexpression induced by cisplatin and enhanced lipid peroxidation [80]. Interestingly, other studies have shown that DNA-damaged RNF113A-deficient cells undergo ferroptosis through the SAT1/ALOX15 signaling pathway [81,82]. Lipopolysaccharide (LPS), a macromolecule composed of lipids and polysaccharides, can cause strong leukocyte-infiltration, free radical production, and induce systemic inflammation [83,84]. In addition, it can disrupt mitochondrial DNA transcription, interfere with the oxidative phosphorylation (OXPHOS) process, reduce the levels of antioxidant enzymes such as glutathione S-transferase (GST) and superoxide dismutase (SOD), thereby inducing ferroptosis in bronchial epithelial cell lines [85,86]. Several studies have demonstrated the involvement of NUPR1 in this LPS-induced ferroptosis. For example, the pancreas showed instantly increased ROS levels and peroxidase enzyme myeloperoxidase (MPO) activity only within the first 6 h after LPS injection in wild-type mice [44]. In NUPR1 knockout mice, however, MPO activity and ROS levels induced by LPS were persistently increased and caused oxidative damage to the pancreas. Interestingly, the kinetics of MPO activity and ROS changes in the lung are the same as those in wild-type mice, indicating a tissue-specificity of NUPR1 in its regulatory function [44]. Furthermore, NUPR1 can transcriptionally regulate the key genes related to ferroptosis resistance, thereby conferring resistance to ferroptosis in cells [2,87,88]. Our latest research also found that by downregulating the expression of GSH-related genes, the NUPR1 inhibitor ZZW-115 affects the antioxidant system and promotes ferroptosis in cancer cells [89].

### 4.2. NUPR1 Controls Iron Metabolism

**Figure 2:**
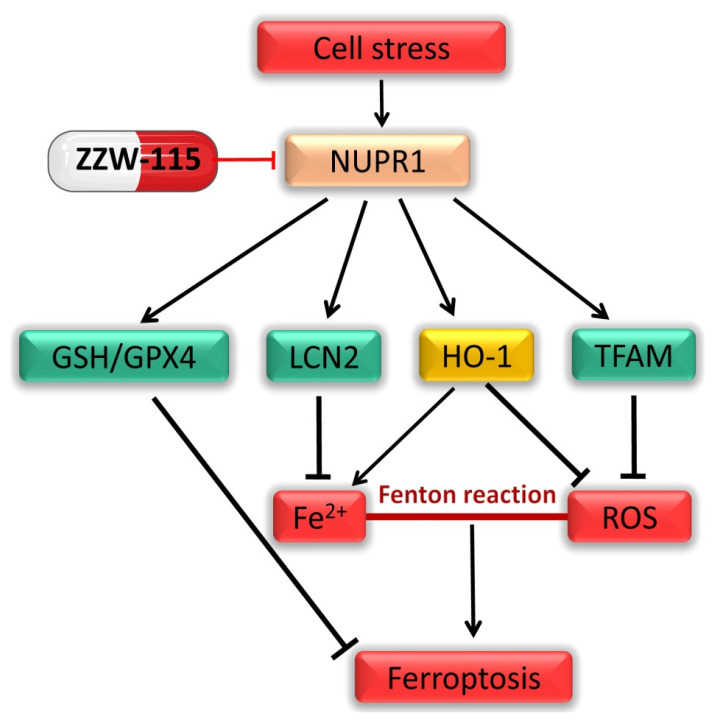
NUPR1 regulates ferroptosis via iron metabolism, ROS homeostasis and the GSH/GPX4 pathway.

In addition to affecting the antioxidant system, NUPR1 also participates in iron metabolism in ferroptosis. It is involved in the regulation of the endogenous enzyme heme oxygenase-1 (HO-1) (Figure 2), which acts to neutralize ROS through its metabolite biliverdin, thus exerting an antioxidant effect [90]. Activation of the Nrf2/HO-1 signaling pathway protects kidney cancer cells from ER stress-related ferroptosis [91]. Similarly, overexpression of HO-1 attenuated the estrogen-mediated ferroptosis in kidney cells, while HO-1 knockout enhanced estrogen-induced ferroptosis in these cells [92]. Consistent with this, H_2_O_2_ dramatically promotes HO-1 reduction because of serum deficiency and induces oxidative damage, accompanied by NUPR1 activation [93]. Targeting NUPR1 prevents this process. Intriguingly, ferrous iron (Fe^2+^), a metabolite of HO-1, can react with intracellular ROS to generate HO•, which causes severe oxidative damage [94]. For example, BAY 11-7085 can induce ferroptosis in liver cancer cells by the overactivation of HO-1 [95]. Knockdown of HO-1 or the utilization of the HO-1-specific inhibitor zinc protoporphyrin IX (ZnPP) can significantly inhibit erastin-induced ferroptosis [96]. Consistent with these results, knockdown of NUPR1 activates HO-1 expression and increases cell viability in keratinocytes and mouse embryonic fibroblast (MEF) cells [97]. The antioxidant properties or pro-oxidant properties of HO-1 in different cells may depend on the balance between ROS and iron [98]. In this context, Liu and colleagues showed in cancer cells that NUPR1 can regulate iron metabolism through transcriptional activation of lipocalin 2 (LCN2) and protect the cells from ferroptosis [29]. Indeed, previous studies have also found that the silencing of NUPR1 down-regulates runt-related transcription factor 2 (RUNX2), a protein that is highly sensitive to iron overload, and ultimately causes premature senescence in breast cancer cells [57]. Therefore, NUPR1 can confer ferroptosis resistance to cancer cells both by enhancing the expression of antioxidant genes and iron metabolism.

### 4.3. NUPR1 Regulates Mitochondrial-Related Ferroptosis

The importance of NUPR1 in maintaining mitochondrial function is now well established, yet the exact role of mitochondria in ferroptosis remains elusive. While the classical ferroptosis inducers such as erastin and RSL3 do not increase mitochondrial ROS, a mitochondrial-targeting antioxidant mitoquinone (MitoQ) can protect against RSL3 toxicity [99]. However, mitochondrial membranes, being as abundant in polyunsaturated fatty acids (PUFAs) as the cell membrane, are also vulnerable to high ROS levels [100]. Indeed, changes in mitochondrial morphology such as condensed mitochondrial membrane densities, reducing mitochondrial volume, and damaged outer membrane are present in the cells treated with ferroptosis inducers [21]. Gao and colleagues found that although mitochondria play an indispensable role in cysteine deficiency-induced ferroptosis, they are dispensable for GPX4 inhibition-induced ferroptosis [101].

A series of recent studies underlined the importance of mitochondria in ferroptosis. The mitochondria-localized antioxidant enzyme dihydroorotate dehydrogenase (DHODH) can prevent oxidative damage to mitochondrial membrane lipids [102]. Its inhibition through brequinar sodium (BQR) induces mitochondrial-related ferroptosis in cancer cells with low expression of GPX4 and enhances the anticancer effect of ferroptosis inducers in cancer cells with a high expression of GPX4 [102]. TFAM is responsible for the replication and transcription of mitochondrial DNA and can reduce ROS production through Lon protease (LONP1) or the down-regulation of nuclear factor of activated T cells (NFAT) [103]. Zalcitabine, an anti-HIV medicine can induce ferroptosis by interfering with the LONP1/TFAM signaling pathway [104,105]. Similarly, our latest research also showed that the NUPR1 inhibitor ZZW-115 can inhibit TFAM expression, cause mitochondrial dysfunction, and produce more ROS production, thereby inducing ferroptosis in a variety of cancer cells such as pancreatic cancer cells and liver cancer cells. Interestingly, ZZW-115-induced ferroptosis can not only be prevented by ferroptosis inhibitors or antioxidants, but also by supplementation with TFAM (our unpublished results) (Figure 2). In summary, NUPR1 inactivation can induce ferroptosis via mitochondrial dysfunction, weakening of antioxidant capacity, and increasing the accumulation of endogenous iron content (Figure 2).

## 5. Conclusions

Regardless of histological type, all tumor cells have excessively high ROS levels and powerful antioxidant systems, which protect against oxidative damage [106,107]. Thus, compared with healthy cells, cancer cells are sensitive to the inhibition of antioxidants [108]. On one hand, as a stress protein, NUPR1 is strongly activated during oxidative stress. On the other hand, as an important transcription factor, NUPR1 can regulate the redox reaction for cancer initiation and development. Inactivation of NUPR1 thus changes multiple intracellular signals and affects cell function; it causes mitochondrial dysfunction, iron metabolic disorder, high ROS production and antioxidant defense impairment, all of which ultimately triggers ferroptosis in cancer cells. Therefore, NUPR1 is a crucial factor in the antioxidant system, and its targeting represents a promising strategy for cancer therapy.

Animal experiments have shown that genetic inactivation of NUPR1 suppresses different types of tumor growth, including pancreatic ductal adenocarcinoma (PDAC) [109], hepatocarcinoma (HCC) [17,110], lung adenocarcinoma [111,112], osteosarcoma [113], glioblastoma [114], cholangiocarcinoma [115], multiple myeloma [116,117,118] and ovarian carcinoma [119]. Among the inhibitors of NUPR1, ZZW-115 shows high potency and better affinity for NUPR1 compared to trifluoperazine (TFP) or other TFP-derived compounds, thus allowing stronger antitumoral activity [62]. The preclinical research demonstrated that ZZW-115 allowed dose-dependent tumor regression in HCC mouse models and different PDAC patient-derived xenograft (PDX) mouse models [62,76]. Importantly, no side effects were observed in these animal models upon ZZW-115 treatment [62]. In addition, NUPR1 can be activated by some anti-cancer agents, thereby conferring drug resistance to the targeted tumors [28]. Depletion of NUPR1 suppressed tumorigenesis and sensitized clear cell renal cell carcinoma to sorafenib treatment in vivo [120]. ZZW-115 treatment enhanced the in vivo anticancer activity of imidazole ketone erastin (IKE) [29] and induced a synergistic effect in combination with either 5-fluorouracil (5-FU) in pancreatic tumors or temozolomide (TMZ) in brain tumors [121]. Altogether, NUPR1 is a promising therapeutic target in cancer therapy. Further studies presenting the development of specific drugs targeting NUPR1 may bring enormous benefits to patients with cancer in the future.

## Abbreviations

NUPR1	Nuclear protein 1
IDP	Intrinsically disordered protein
ER	Endoplasmic reticulum
ROS	Reactive oxygen species
KRAS	V-Ki-ras2 Kirsten rat sarcoma viral oncogene homolog
NAC	N-acetyl cysteine
Cd	Cadmium
GSH	Glutathione
METH	Methamphetamine
mTOR	Mechanistic target of rapamycin
UPS	Ubiquitin-proteasome system
PDAC	Pancreas ductal adenocarcinoma
HCC	Hepatocarcinoma
TFP	Trifluoperazine
PDX	Patient-derived xenograft
IKE	Imidazole ketone erastin
5-FU	5-fluorouracil
TMZ	Temozolomide
UPR	Unfolded protein response
TBHP	Tert-butyl hydroperoxide
ATF3	Activating transcription factor 3
eIF2α	Eukaryotic initiation factor 2α
GRP75	Glucose-regulated protein 75
GRP94	Glucose-regulated protein 94
H_2_O_2_	Hydrogen peroxide
AP-1	Activator protein 1
Ni	Nickel
Ca^2+^	Calcium ions
Fe^2+^	Ferrous iron
HO•	Hydroxyl radical
Nrf2	Nuclear factor erythroid 2–related factor 2
AKR1C1	Aldo-keto reductase family 1 member C1
SNAP25	Synaptosome Associated Protein 25
MMP	Mitochondrial membrane potential
TCA	Tricarboxylic acid cycle
AURKA	Aurora kinase A
Nox1	NADPH oxidase 1
DHODH	dihydroorotate dehydrogenase
BQR	Brequinar sodium
Rac1	Ras-related C3 botulinum toxin substrate 1
PGC-1α	Coactivator 1-alpha
FAS	Fatty acid synthase
HFD	High-fat diet
LPS	Lipopolysaccharide
OXPHOS	Oxidative phosphorylation
GST	Glutathione S-transferase
SOD	Superoxide dismutase
MPO	Myeloperoxidase
HO-1	Heme oxygenase-1
MEF	Mouse embryonic fibroblasts
ZnPP	Zinc protoporphyrin IX
LCN2	Lipocalin 2
RUNX2	Runt-related transcription factor 2
MitoQ	Mitoquinone
PUFAs	Polyunsaturated fatty acids
TFAM	Mitochondrial transcription factor A
LONP1	Lon protease
NFAT	Nuclear factor of activated T cells

## References

[1] Pommier R., Gout J., Vincent D.F., Cano C.E., Kaniewski B., Martel S., Rodriguez J., Fourel G., Valcourt U., Marie J. (2012). The human NUPR1/P8 gene is transcriptionally activated by transforming growth factor β via the SMAD signalling pathway. Biochem. J..

[2] Cano C.E., Hamidi T., Sandi M.J., Iovanna J. (2011). Nupr1: The Swiss-knife of cancer. J. Cell. Physiol..

[3] Giampietri C., Petrungaro S., Conti S., Facchiano A., Filippini A., Ziparo E. (2015). Cancer Microenvironment and Endoplasmic Reticulum Stress Response. Mediat. Inflamm..

[4] Avril T., Vauléon E., Chevet E. (2017). Endoplasmic reticulum stress signaling and chemotherapy resistance in solid cancers. Oncogenesis.

[5] Santofimia-Castaño P., Rizzuti B., Xia Y., Abian O., Peng L., Velazquez-Campoy A., Neira J.L., Iovanna J. (2020). Targeting intrinsically disordered proteins involved in cancer. Cell. Mol. Life Sci..

[6] Schieber M., Chandel N.S. (2014). ROS Function in Redox Signaling and Oxidative Stress. Curr. Biol..

[7] Idelchik M.D.P.S., Begley U., Begley T.J., Melendez J.A. (2017). Mitochondrial ROS control of cancer. Semin. Cancer Biol..

[8] Qu J., Zou T., Lin Z. (2021). The Roles of the Ubiquitin–Proteasome System in the Endoplasmic Reticulum Stress Pathway. Int. J. Mol. Sci..

[9] Gardner B.M., Pincus D., Gotthardt K., Gallagher C.M., Walter P. (2013). Endoplasmic Reticulum Stress Sensing in the Unfolded Protein Response. Cold Spring Harb. Perspect. Biol..

[10] Yadav R.K., Chae S.-W., Kim H.-R., Chae H.J. (2014). Endoplasmic Reticulum Stress and Cancer. J. Cancer Prev..

[11] Xu X., Huang E., Tai Y., Zhao X., Chen X., Chen C., Chen R., Liu C., Lin Z., Wang H. (2017). Nupr1 Modulates Methamphetamine-Induced Dopaminergic Neuronal Apoptosis and Autophagy through CHOP-Trib3-Mediated Endoplasmic Reticulum Stress Signaling Pathway. Front. Mol. Neurosci..

[12] Cai D., Huang E., Luo B., Yang Y., Zhang F., Liu C., Lin Z., Xie W.-B., Wang H. (2016). Nupr1/Chop signal axis is involved in mitochondrion-related endothelial cell apoptosis induced by methamphetamine. Cell Death Dis..

[13] Xu X., Huang E., Luo B., Cai D., Zhao X., Luo Q., Jin Y., Chen L., Wang Q., Liu C. (2018). Methamphetamine exposure triggers apoptosis and autophagy in neuronal cells by activating the C/EBPβ-related signaling pathway. FASEB J..

[14] Cao S.S., Kaufman R.J. (2014). Endoplasmic Reticulum Stress and Oxidative Stress in Cell Fate Decision and Human Disease. Antioxid. Redox Signal..

[15] Kim J., Bae J.-S. (2016). ROS homeostasis and metabolism: A critical liaison for cancer therapy. Exp. Mol. Med..

[16] Wang L., Sun J., Yin Y., Sun Y., Ma J., Zhou R., Chang X., Li D., Yao Z., Tian S. (2021). Transcriptional coregualtor NUPR1 maintains tamoxifen resistance in breast cancer cells. Cell Death Dis..

[17] Emma M.R., Iovanna J.L., Bachvarov D., Puleio R., Loria G.R., Augello G., Candido S., Libra M., Gulino A., Cancila V. (2016). NUPR1, a new target in liver cancer: Implication in controlling cell growth, migration, invasion and sorafenib resistance. Cell Death Dis..

[18] Dixon S.J., Lemberg K.M., Lamprecht M.R., Skouta R., Zaitsev E.M., Gleason C.E., Patel D.N., Bauer A.J., Cantley A.M., Yang W.S. (2012). Ferroptosis: An Iron-Dependent Form of Nonapoptotic Cell Death. Cell.

[19] Kapralov O., Yang Q., Dar H.H., Tyurina Y., Anthonymuthu T.S., Kim R., Croix C.M.S., Mikulska-Ruminska K., Liu B., Shrivastava I.H. (2020). Redox lipid reprogramming commands susceptibility of macrophages and microglia to ferroptotic death. Nat. Chem. Biol..

[20] Zhu J., Xiong Y., Zhang Y., Wen J., Cai N., Cheng K., Liang H., Zhang W. (2020). The Molecular Mechanisms of Regulating Oxidative Stress-Induced Ferroptosis and Therapeutic Strategy in Tumors. Oxid. Med. Cell. Longev..

[21] Li J., Cao F., Yin H.-L., Huang Z.-J., Lin Z.-T., Mao N., Sun B., Wang G. (2020). Ferroptosis: Past, present and future. Cell Death Dis..

[22] Yang W.S., SriRamaratnam R., Welsch M.E., Shimada K., Skouta R., Viswanathan V., Cheah J.H., Clemons P.A., Shamji A.F., Clish C. (2014). Regulation of Ferroptotic Cancer Cell Death by GPX4. Cell.

[23] Doll S., Freitas F.P., Shah R., Aldrovandi M., da Silva M.C., Ingold I., Grocin A.G., da Silva T.N.X., Panzilius E., Scheel C.H. (2019). FSP1 is a glutathione-independent ferroptosis suppressor. Nat. Cell Biol..

[24] Bersuker K., Hendricks J.M., Li Z., Magtanong L., Ford B., Tang P.H., Roberts M.A., Tong B., Maimone T.J., Zoncu R. (2019). The CoQ oxidoreductase FSP1 acts parallel to GPX4 to inhibit ferroptosis. Nat. Cell Biol..

[25] Soula M., Weber R.A., Zilka O., Alwaseem H., La K., Yen F., Molina H., Garcia-Bermudez J., Pratt D.A., Birsoy K. (2020). Metabolic determinants of cancer cell sensitivity to canonical ferroptosis inducers. Nat. Chem. Biol..

[26] Jiang X., Stockwell B.R., Conrad M. (2021). Ferroptosis: Mechanisms, biology and role in disease. Nat. Rev. Mol. Cell Biol..

[27] Lin X., Ping J., Wen Y., Wu Y. (2020). The Mechanism of Ferroptosis and Applications in Tumor Treatment. Front. Pharmacol..

[28] Murphy A., Costa M. (2020). Nuclear protein 1 imparts oncogenic potential and chemotherapeutic resistance in cancer. Cancer Lett..

[29] Liu J., Song X., Kuang F., Zhang Q., Xie Y., Kang R., Kroemer G., Tang D. (2021). NUPR1 is a critical repressor of ferroptosis. Nat. Commun..

[30] Hamidi T., Cano C.E., Grasso D., Garcia M.N., Sandí M.J., Calvo E.L., Dagorn J.-C., Lomberk G., Goruppi S., Urrutia R. (2013). NUPR1 works against the metabolic stress-induced autophagy-associated cell death in pancreatic cancer cells. Autophagy.

[31] Wu B., Zeng W., Ouyang W., Xu Q., Chen J., Wang B., Zhang X. (2020). Quercetin induced NUPR1-dependent autophagic cell death by disturbing reactive oxygen species homeostasis in osteosarcoma cells. J. Clin. Biochem. Nutr..

[32] Bolduc J.-S., Denizeau F., Jumarie C. (2004). Cadmium-Induced Mitochondrial Membrane-Potential Dissipation Does Not Necessarily Require Cytosolic Oxidative Stress: Studies Using Rhodamine-123 Fluorescence Unquenching. Toxicol. Sci..

[33] Patra R.C., Rautray A.K., Swarup D. (2011). Oxidative Stress in Lead and Cadmium Toxicity and Its Amelioration. SAGE-Hindawi Access Res..

[34] Fan T., Chen Y., He Z., Wang Q., Yang X., Ren Z., Zhang S. (2019). Inhibition of ROS/NUPR1-dependent autophagy antagonises repeated cadmium exposure -induced oral squamous cell carcinoma cell migration and invasion. Toxicol. Lett..

[35] Bhattarai K.R., Alam Riaz T., Kim H.-R., Chae H.-J. (2021). The aftermath of the interplay between the endoplasmic reticulum stress response and redox signaling. Exp. Mol. Med..

[36] Chen X., Yu C., Kang R., Kroemer G., Tang D. (2021). Cellular degradation systems in ferroptosis. Cell Death Differ..

[37] Zeeshan H.M.A., Lee G.H., Kim H.-R., Chae H.-J. (2016). Endoplasmic Reticulum Stress and Associated ROS. Int. J. Mol. Sci..

[38] Bhandary B., Marahatta A., Kim H.-R., Chae H.-J. (2012). An Involvement of Oxidative Stress in Endoplasmic Reticulum Stress and Its Associated Diseases. Int. J. Mol. Sci..

[39] Abdullah C.S., Aishwarya R., Alam S., Morshed M., Remex N.S., Nitu S., Kolluru G.K., Traylor J., Miriyala S., Panchatcharam M. (2020). Methamphetamine induces cardiomyopathy by Sigmar1 inhibition-dependent impairment of mitochondrial dynamics and function. Commun. Biol..

[40] Lee Y.-K., Jee B.A., Kwon S.M., Yoon Y.-S., Xu W.G., Wang H.-J., Wang X.W., Thorgeirsson S.S., Lee J.-S., Woo H.G. (2015). Identification of a mitochondrial defect gene signature reveals NUPR1 as a key regulator of liver cancer progression. Hepatology.

[41] Okamoto A., Iwamoto Y., Maru Y. (2006). Oxidative Stress-Responsive Transcription Factor ATF3 Potentially Mediates Diabetic Angiopathy. Mol. Cell. Biol..

[42] Yammani R.R., Loeser R.F. (2014). Brief report: Stress-inducible nuclear protein 1 regulates matrix metalloproteinase 13 expression in human articular chondrocytes. Arth. Rheumatol..

[43] Wenz C., Faust D., Linz B., Turmann C., Nikolova T., Bertin J., Gough P., Wipf P., Schroder A.S., Krautwald S. (2018). t-BuOOH induces ferroptosis in human and murine cell lines. Arch. Toxicol..

[44] Vasseur S., Hoffmeister A., Garcia-Montero A., Barthet M., Saint-Michel L., Berthézène P., Fiedler F., Closa D., Dagorn J.C., Iovanna J.L. (2003). Mice with targeted disruption of p8gene show increased sensitivity to lipopolysaccharide and DNA microarray analysis of livers reveals an aberrant gene expression response. BMC Gastroenterol..

[45] Murphy A., Roy N., Sun H., Jin C., Costa M. (2021). Induction of NUPR1 and AP-1 contributes to the carcinogenic potential of nickel. Oncol. Rep..

[46] Hamidi T., Cano C.E., Grasso D., Garcia M.N., Sandi M.J., Calvo E.L., Dagorn J.-C., Lomberk G., Urrutia R., Goruppi S. (2012). Nupr1-Aurora Kinase A Pathway Provides Protection against Metabolic Stress-Mediated Autophagic-Associated Cell Death. Clin. Cancer Res..

[47] Kang M.A., So E.-Y., Simons A.L., Spitz D., Ouchi T. (2012). DNA damage induces reactive oxygen species generation through the H2AX-Nox1/Rac1 pathway. Cell Death Dis..

[48] Barbosa-Sampaio H.C., Drynda R., Liu B., De Ledesma A.R., Malicet C., Iovanna J., Jones P., Muller D., Persaud S. (2015). Reduced nuclear protein 1 expression improves insulin sensitivity and protects against diet-induced glucose intolerance through up-regulation of heat shock protein 70. Biochim. Biophys. Acta Mol. Basis Dis..

[49] Ko E.-J., Ock M.-S., Choi Y.-H., Iovanna J., Mun S., Han K., Kim H.-S., Cha H.-J. (2021). Human Endogenous Retrovirus (HERV)-K *env* Gene Knockout Affects Tumorigenic Characteristics of *nupr1* Gene in DLD-1 Colorectal Cancer Cells. Int. J. Mol. Sci..

[50] Pierre N., Barbé C., Gilson H., Deldicque L., Raymackers J.-M., Francaux M. (2014). Activation of ER stress by hydrogen peroxide in C_2_C_12_ myotubes. Biochem. Biophys. Res. Commun..

[51] Borrello M.T., Santofimia-Castaño P., Bocchio M., Listi A., Fraunhoffer N., Soubeyran P., Chevet E., Pin C., Iovanna J. (2021). NUPR1 interacts with eIF2α and is required for resolution of the ER stress response in pancreatic tissue. FEBS J..

[52] Santofimia-Castaño P., Lan W., Bintz J., Gayet O., Carrier A., Lomberk G., Neira J.L., González A., Urrutia R., Soubeyran P. (2018). Inactivation of NUPR1 promotes cell death by coupling ER-stress responses with necrosis. Sci. Rep..

[53] Uppala R., McKinney R.W., Brant K.A., Fabisiak J.P., Goetzman E.S. (2015). Nickel inhibits mitochondrial fatty acid oxidation. Biochem. Biophys. Res. Commun..

[54] Ma Q. (2013). Role of Nrf2 in Oxidative Stress and Toxicity. Annu. Rev. Pharmacol. Toxicol..

[55] He F., Ru X., Wen T. (2020). NRF2, a Transcription Factor for Stress Response and Beyond. Int. J. Mol. Sci..

[56] Narzt M.-S., Nagelreiter I.M., Oskolkova O., Bochkov V.N., Latreille J., Fedorova M., Ni Z., Sialana F., Lubec G., Filzwieser M. (2019). A novel role for NUPR1 in the keratinocyte stress response to UV oxidized phospholipids. Redox Biol..

[57] Mu Y., Yan X., Li D., Zhao D., Wang L., Wang X., Gao D., Yang J., Zhang H., Li Y. (2018). NUPR1 maintains autolysosomal efflux by activating SNAP25 transcription in cancer cells. Autophagy.

[58] Giniatullin A., Darios F., Shakirzyanova A., Davletov B. (2006). SNAP25 is a pre-synaptic target for the depressant action of reactive oxygen species on transmitter release. J. Neurochem..

[59] Porporato P.E., Filigheddu N., Pedro J.M.B.-S., Kroemer G., Galluzzi L. (2018). Mitochondrial metabolism and cancer. Cell Res..

[60] Anatoliotakis N., Deftereos S., Bouras G., Giannopoulos G., Tsounis D., Angelidis C., Kaoukis A., Stefanadis C. (2013). Myeloperoxidase: Expressing Inflammation and Oxidative Stress in Cardiovascular Disease. Curr. Top. Med. Chem..

[61] Meng N., Mu X., Lv X., Wang L., Li N., Gong Y. (2019). Autophagy represses fascaplysin-induced apoptosis and angiogenesis inhibition via ROS and p8 in vascular endothelia cells. Biomed. Pharmacother..

[62] Santofimia-Castaño P., Xia Y., Lan W., Zhou Z., Huang C., Peng L., Soubeyran P., Velazquez-Campoy A., Abián O., Rizzuti B. (2019). Ligand-based design identifies a potent NUPR1 inhibitor exerting anticancer activity via necroptosis. J. Clin. Investig..

[63] Spinelli J.B., Haigis M.C. (2018). The multifaceted contributions of mitochondria to cellular metabolism. Nat. Cell Biol..

[64] Aguado-Llera D., Hamidi T., Doménech R., Pantoja-Uceda D., Gironella M., Santoro J., Velazquez-Campoy A., Neira J.L., Iovanna J.L. (2013). Deciphering the Binding between Nupr1 and MSL1 and Their DNA-Repairing Activity. PLoS ONE.

[65] Gomaa A., Peng D., Chen Z., Soutto M., Abouelezz K., Corvalan A., El-Rifai W. (2019). Epigenetic regulation of AURKA by miR-4715-3p in upper gastrointestinal cancers. Sci. Rep..

[66] Xie Y., Zhu S., Zhong M., Yang M., Sun X., Liu J., Kroemer G., Lotze M., Zeh H.J., Kang R. (2017). Inhibition of Aurora Kinase A Induces Necroptosis in Pancreatic Carcinoma. Gastroenterology.

[67] Pouysségur J., Shiu R.P., Pastan I. (1977). Induction of two transformation-sensitive membrane polypeptides in normal fibroblasts by a block in glycoprotein synthesis or glucose deprivation. Cell.

[68] Chen S.-D., Yang D.-I., Lin T.-K., Shaw F.-Z., Liou C.-W., Chuang Y.-C. (2011). Roles of Oxidative Stress, Apoptosis, PGC-1α and Mitochondrial Biogenesis in Cerebral Ischemia. Int. J. Mol. Sci..

[69] Onishi Y., Ueha T., Kawamoto T., Hara H., Toda M., Harada R., Minoda M., Kurosaka M., Akisue T. (2014). Regulation of Mitochondrial Proliferation by PGC-1α Induces Cellular Apoptosis in Musculoskeletal Malignancies. Sci. Rep..

[70] Jiang W.G., Davies G., Kynaston H., Mason M.D., Fodstad O. (2006). Does the PGC-1/PPARgamma pathway play a role in Com-1/p8 mediated cell growth inhibition in prostate cancer?. Int. J. Mol. Med..

[71] Hollenbach M., Klöting N., Sommerer I., Lorenz J., Heindl M., Kern M., Mossner J., Bluher M., Hoffmeister A. (2018). p8 deficiency leads to elevated pancreatic beta cell mass but does not contribute to insulin resistance in mice fed with high-fat diet. PLoS ONE.

[72] Li Z., Rasmussen M.L., Li J., Henríquez-Olguín C., Knudsen J.R., Madsen A.B., Quant E.S.S., Kleinert M., Jensen T.E. (2018). Periodized low protein-high carbohydrate diet confers potent, but transient, metabolic improvements. Mol. Metab..

[73] Päth G., Opel A., Knoll A., Seufert J. (2004). Nuclear Protein p8 Is Associated with Glucose-Induced Pancreatic β-Cell Growth. Diabetes.

[74] Zhou D., Shao L., Spitz D.R. (2014). Reactive oxygen species in normal and tumor stem cells. Adv. Cancer Res..

[75] Kumari S., Badana A.K., Murali Mohan G., Shailender G., Malla R.R. (2018). Reactive Oxygen Species: A Key Constituent in Cancer Survival. Biomark. Insights.

[76] Lan W., Santofimia-Castaño P., Xia Y., Zhou Z., Huang C., Fraunhoffer N., Barea D., Cervello M., Giannitrapani L., Montalto G. (2020). Targeting NUPR1 with the small compound ZZW-115 is an efficient strategy to treat hepatocellular carcinoma. Cancer Lett..

[77] Santofimia-Castaño P., Iovanna J. (2021). Combating pancreatic cancer chemoresistance by triggering multiple cell death pathways. Pancreatol..

[78] Miura G. (2021). A recipe for execution. Nat. Chem. Biol..

[79] He Y.-J., Liu X.-Y., Xing L., Wan X., Chang X., Jiang H.-L. (2020). Fenton reaction-independent ferroptosis therapy via glutathione and iron redox couple sequentially triggered lipid peroxide generator. Biomaterials.

[80] Shostak K., Jiang Z., Charloteaux B., Mayer A., Habraken Y., Tharun L., Klein S., Xu X., Duong H.Q., Vislovukh A. (2020). The X-linked trichothiodystrophy-causing gene RNF113A links the spliceosome to cell survival upon DNA damage. Nat. Commun..

[81] Ou Y., Wang S.-J., Li D., Chu B., Gu W. (2016). Activation of SAT1 engages polyamine metabolism with p53-mediated ferroptotic responses. Proc. Natl. Acad. Sci. USA.

[82] Tang D., Chen X., Kang R., Kroemer G. (2021). Ferroptosis: Molecular mechanisms and health implications. Cell Res..

[83] Ahmadabady S., Beheshti F., Shahidpour F., Khordad E., Hosseini M. (2021). A protective effect of curcumin on cardiovascular oxidative stress indicators in systemic inflammation induced by lipopolysaccharide in rats. Biochem. Biophys. Rep..

[84] Saji N., Francis N., Schwarz L.J., Blanchard C.L., Santhakumar A.B. (2020). The Antioxidant and Anti-Inflammatory Properties of Rice Bran Phenolic Extracts. Foods.

[85] Li J., Lu K., Sun F., Tan S., Zhang X., Sheng W., Hao W., Liu M., Lv W., Han W. (2021). Panaxydol attenuates ferroptosis against LPS-induced acute lung injury in mice by Keap1-Nrf2/HO-1 pathway. J. Transl. Med..

[86] Kuwabara T., Imajoh-Ohmi S. (2004). LPS-induced apoptosis is dependent upon mitochondrial dysfunction. Apoptosis.

[87] Jin H.-O., Seo S.-K., Woo S.-H., Choe T.-B., Hong S.-I., Kim J.-I., Park I.-C. (2009). Nuclear protein 1 induced by ATF4 in response to various stressors acts as a positive regulator on the transcriptional activation of ATF4. IUBMB Life.

[88] Rozpedek W., Pytel D., Mucha B., Leszczynska H., Diehl J.A., Majsterek I. (2016). The Role of the PERK/eIF2α/ATF4/CHOP Signaling Pathway in Tumor Progression During Endoplasmic Reticulum Stress. Curr. Mol. Med..

[89] Huang C., Santofimia-Castaño P., Lan W., Fraunhoffer N., Meilerman A., Iovanna J. (2020). Inducing ferroptosis by the NUPR1 inhibitor ZZW115 to kill pancreatic cancer cells. Pancreatology.

[90] Bauer M., Bauer I. (2002). Heme Oxygenase-1: Redox Regulation and Role in the Hepatic Response to Oxidative Stress. Antioxid. Redox Signal..

[91] Deng H.-F., Yue L.-X., Wang N.-N., Zhou Y.-Q., Zhou W., Liu X., Ni Y.-H., Huang C.-S., Qiu L.-Z., Liu H. (2021). Mitochondrial Iron Overload-Mediated Inhibition of Nrf2-HO-1/GPX4 Assisted ALI-Induced Nephrotoxicity. Front. Pharmacol..

[92] Li B., Yang L., Peng X., Fan Q., Wei S., Yang S., Li X., Jin H., Wu B., Huang M. (2020). Emerging mechanisms and applications of ferroptosis in the treatment of resistant cancers. Biomed. Pharmacother..

[93] Carracedo A., Egia A., Guzman M., Velasco G. (2006). p8 Upregulation sensitizes astrocytes to oxidative stress. FEBS Lett..

[94] Araujo J.A., Zhang M., Yin F. (2012). Heme Oxygenase-1, Oxidation, Inflammation, and Atherosclerosis. Front. Pharmacol..

[95] Chang L.-C., Chiang S.-K., Chen S.-E., Yu Y.-L., Chou R.-H., Chang W.-C. (2018). Heme oxygenase-1 mediates BAY 11–7085 induced ferroptosis. Cancer Lett..

[96] Fang X., Wang H., Han D., Xie E., Yang X., Wei J., Gu S., Gao F., Zhu N., Yin X. (2019). Ferroptosis as a target for protection against cardiomyopathy. Proc. Natl. Acad. Sci. USA.

[97] Weis S., Bielow T., Sommerer I., Iovanna J., Malicet C., Mössner J., Hoffmeister A. (2015). P8 deficiency increases cellular ROS and induces HO-1. Arch. Biochem. Biophys..

[98] Chiang S.-K., Chen S.-E., Chang L.-C. (2018). A Dual Role of Heme Oxygenase-1 in Cancer Cells. Int. J. Mol. Sci..

[99] Jelinek A., Heyder L., Daude M., Plessner M., Krippner S., Grosse R., Diederich W.E., Culmsee C. (2018). Mitochondrial rescue prevents glutathione peroxidase-dependent ferroptosis. Free. Radic. Biol. Med..

[100] Sullivan E.M., Pennington E.R., Green W.D., A Beck M., A Brown D., Shaikh S.R. (2018). Mechanisms by Which Dietary Fatty Acids Regulate Mitochondrial Structure-Function in Health and Disease. Adv. Nutr..

[101] Gao M., Yi J., Zhu J., Minikes A., Monian P., Thompson C.B., Jiang X. (2019). Role of Mitochondria in Ferroptosis. Mol. Cell.

[102] Garcia-Bermudez J., Birsoy K. (2021). A mitochondrial gatekeeper that helps cells escape death by ferroptosis. Nat. Cell Biol..

[103] Kunkel G.H., Chaturvedi P., Tyagi S.C. (2016). Mitochondrial pathways to cardiac recovery: TFAM. Hear. Fail. Rev..

[104] Li C., Zhang Y., Liu J., Kang R., Klionsky D.J., Tang D. (2021). Mitochondrial DNA stress triggers autophagy-dependent ferroptotic death. Autophagy.

[105] Chen X., Kang R., Kroemer G., Tang D. (2021). Broadening horizons: The role of ferroptosis in cancer. Nat. Rev. Clin. Oncol..

[106] Reczek C.R., Chandel N.S. (2017). The Two Faces of Reactive Oxygen Species in Cancer. Annu. Rev. Cancer Biol..

[107] Perillo B., Di Donato M., Pezone A., Di Zazzo E., Giovannelli P., Galasso G., Castoria G., Migliaccio A. (2020). ROS in cancer therapy: The bright side of the moon. Exp. Mol. Med..

[108] Harris I., DeNicola G.M. (2020). The Complex Interplay between Antioxidants and ROS in Cancer. Trends Cell Biol..

[109] Sandi M.J., Hamidi T., Malicet C., Cano C., Loncle C., Pierres A., Dagorn J.C., Iovanna J.L. (2011). p8 Expression controls pancreatic cancer cell migration, invasion, adhesion, and tumorigenesis. J. Cell. Physiol..

[110] Chen C.-Y., Wu S.-M., Lin Y.-H., Chi H.-C., Lin S.-L., Yeh C.-T., Chuang W.-Y., Lin K.-H. (2019). Induction of nuclear protein-1 by thyroid hormone enhances platelet-derived growth factor A mediated angiogenesis in liver cancer. Theranostics.

[111] Guo X., Wang W., Hu J., Feng K., Pan Y., Zhang L., Feng Y. (2012). Lentivirus-mediated RNAi knockdown of NUPR1 inhibits human nonsmall cell lung cancer growth in vitro and in vivo. Anat. Rec. Adv. Integr. Anat. Evol. Biol..

[112] Li Y., Yin Y., Ma J., Sun Y., Zhou R., Cui B., Zhang Y., Yang J., Yan X., Liu Z. (2020). Combination of AAV-mediated NUPR1 knockdown and trifluoperazine induces premature senescence in human lung adenocarcinoma A549 cells in nude mice. Oncol. Rep..

[113] Zhou C., Xu J., Lin J., Lin R., Chen K., Kong J., Shui X. (2018). Long Noncoding RNA FEZF1-AS1 Promotes Osteosarcoma Progression by Regulating the miR-4443/NUPR1 Axis. Oncol. Res. Featur. Preclin. Clin. Cancer Ther..

[114] Li J., Ren S., Liu Y., Lian Z., Dong B., Yao Y., Xu Y. (2017). Knockdown of NUPR1 inhibits the proliferation of glioblastoma cells via ERK1/2, p38 MAPK and caspase-3. J. Neuro-Oncol..

[115] Kim K.-S., Jin D.-I., Yoon S., Baek S.-Y., Kim B.-S., Oh S.-O. (2012). Expression and roles of NUPR1 in cholangiocarcinoma cells. Anat. Cell Biol..

[116] Zeng C., Yi B., Li X., Chen J. (2017). Knockdown of nuclear protein 1 (NUPR1) gene inhibits proliferation and promotes apoptosis of human multiple myeloma U266 cells. Chin. J. Cell. Mol. Immunol..

[117] Zeng C., Li X., Li A., Yi B., Peng X., Huang X., Chen J. (2018). Knockdown of NUPR1 inhibits the growth of U266 and RPMI8226 multiple myeloma cell lines via activating PTEN and caspase activation-dependent apoptosis. Oncol. Rep..

[118] Li A., Li X., Chen X., Zeng C., Wang Z., Li Z., Chen J. (2020). NUPR1 Silencing Induces Autophagy-Mediated Apoptosis in Multiple Myeloma Cells Through the PI3K/AKT/mTOR Pathway. DNA Cell Biol..

[119] Yu J., Zhu H., Li R., Jiang Q., Luan W., Shi J., Liu P. (2020). Oncogenic Role of NUPR1 in Ovarian Cancer. Oncotargets Ther..

[120] He W., Cheng F., Zheng B., Wang J., Zhao G., Yao Z., Zhang T. (2021). NUPR1 is a novel potential biomarker and confers resistance to sorafenib in clear cell renal cell carcinoma by increasing stemness and targeting the PTEN/AKT/mTOR pathway. Aging.

[121] Lan W., Santofimia-Castaño P., Swayden M., Xia Y., Zhou Z., Audebert S., Camoin L., Huang C., Peng L., Jiménez-Alesanco A. (2020). ZZW-115–dependent inhibition of NUPR1 nuclear translocation sensitizes cancer cells to genotoxic agents. JCI Insight.

